# Predicting Short-term Survival after Liver Transplantation using Machine Learning

**DOI:** 10.1038/s41598-020-62387-z

**Published:** 2020-03-27

**Authors:** Chien-Liang Liu, Ruey-Shyang Soong, Wei-Chen Lee, Guo-Wei Jiang, Yun-Chun Lin

**Affiliations:** 1 Department of Industrial Engineering and Management, National Chiao Tung University, Hsinchu, 300 Taiwan R.O.C.; 2 Department of General Surgery, Chang Gung Memorial Hospital, Keelung, Taiwan R.O.C.; 3 Laboratory of Immunology, Department of General Surgery, Chang Gung Memorial Hospital, Taipei, Taiwan R.O.C.; 4 Chang Gung University College of Medicine, Taoyuan, Taiwan R.O.C.

**Keywords:** Liver, Outcomes research

## Abstract

Liver transplantation is one of the most effective treatments for end-stage liver disease, but the demand for livers is much higher than the available donor livers. Model for End-stage Liver Disease (MELD) score is a commonly used approach to prioritize patients, but previous studies have indicated that MELD score may fail to predict well for the postoperative patients. This work proposes to use data-driven approach to devise a predictive model to predict postoperative survival within 30 days based on patient’s preoperative physiological measurement values. We use random forest (RF) to select important features, including clinically used features and new features discovered from physiological measurement values. Moreover, we propose a new imputation method to deal with the problem of missing values and the results show that it outperforms the other alternatives. In the predictive model, we use patients’ blood test data within 1–9 days before surgery to construct the model to predict postoperative patients’ survival. The experimental results on a real data set indicate that RF outperforms the other alternatives. The experimental results on the temporal validation set show that our proposed model achieves area under the curve (AUC) of 0.771 and specificity of 0.815, showing superior discrimination power in predicting postoperative survival.

## Introduction

Liver transplantation is one of the most effective treatments in treating acute liver failure, chronic liver cirrhosis and even hepatocellular carcinomas^1^. Unfortunately, the demand for liver is much higher than the number of available donor livers, explaining why patients on the waiting list for a liver transplantation have to be prioritized. End-Stage Liver Disease (MELD) score^2–4^ has been widely used to estimate the severity of liver disease in patients^5^, and has been accepted as a standard criterion of the allocation of liver graft worldwide. Although high MELDs are presumed to link to the worse outcome, but most of the studies failed to show the accurate prediction rate between pre-transplantation MELDs and the post-transplantation survival outcome^6,7^. Therefore, a promising model that can predict the survival outcome after liver transplantation is essential in liver transplantation. Although several predictive models of postoperative survival for liver transplantation have been proposed in the past decades^8–12^, using machine learning to construct predictive model is still limited. This work focuses on constructing a model for the prediction of postoperative survival to help physicians make decisions.

With the advancement of electronic medical record and hospital information system, hospitals have increasingly stored patient information in electronic format and it is possible to use these records to discover patterns in the data^13,14^. To analyze these data records, more and more researchers have applied machine learning to medical fields such as disease diagnosis^15–17^, early warning systems^18–20^, and drug discovery^21–23^. In the past, researchers tended to use statistical methods such as regression or cox regression to predict postoperative survival, but the prediction performances are not good enough to be applied to clinical cases. This work proposes to use machine learning to develop a prediction model for the postoperative survival of liver transplantation, since machine learning does not assume the distribution of the underlying data and many state-of-the-art algorithms have been devised over the decades^24–26^. For example, a previous study used classification trees to predict a candidate’s 3-month wait-list mortality with Standard Transplant Analysis and Research (STAR) data set, providing more accurate and objective predictions than MELD in prioritizing candidates for liver transplantation^27^. Moreover, the previous study indicated that the model with selected features from RF achieved excellent performance in predicting graft failure^28^. Notably, this work is different from these works as we only use patient basic information such as body mass index (BMI), age, and blood test data within 1–9 days before surgery to develop the model. It is worth mentioning that these features are easily accessible, and our model focuses on short-term survival after the surgery of liver transplantation. Moreover, machine learning enables the system to learn from data, giving a base to build and constantly refine a model for making accurate predictions.

The objective of this research is to use data-driven technique to develop a predictive model to predict postoperative survival within 30 days for the patients who have undergone liver transplantation. To construct and validate the proposed model, we use machine learning along with real data form liver transplantation Intensive Care Unit (ICU) of Chang Gung Memorial Hospital, Linkou which range from January, 2004 to December, 2013. Note that the prediction in this work is a binary classification problem rather than a survival analysis as the purpose is to predict postoperative survival with the outcome to be either "Survival” or "Non-survival” that is defined by whether the survival time of a patient is more than 30 days. To validate our proposed model, we used the patients who received the surgery during 2004 and 2012 as the derivation set, while the patient data after 2013 were used as the temporal validation set. Notably, we developed the prediction with the derivation set, and validated the model with temporal validation set.

## Results

Random forest^29^ (RF) is a state-of-the-art algorithm, and it could provide feature importance based on the out-of-bag samples and permutation test, in which informative variables produce a systematic decrease in accuracy when permuted. This work uses RF to estimate the feature importance, and the top nine important features are international normalized ratio (INR), lymphocytes, prothrombin time (PT), platelets, white blood cell (WBC), Magnesium (Mg), Sodium (Na), age, and BMI. The PT and INR represent the same measurement, so we only use INR to construct the model in the following experiments to prevent from bias brought by duplicated variables.

To evaluate model performance, we use the AUC as the evaluation metric. The AUC provides the overall result of the receiver operating characteristic (ROC) curve by using the area under the ROC curve as an important metric for evaluating the predictive model. The value of AUC is between 0 and 1, and a larger value indicates that the classifier yields better performance. ROC curve is commonly used in the medical field to determine thresholds for patient diagnosis. More detailed introduction about ROC and AUC can refer to the work conducted by Fawcett^30^. Besides AUC, sensitivity and specificity are also used as the metrics. Notably, specificity is more important than sensitivity in this work as present study aims to predict survival outcome after the surgery of liver transplantation.

In order to explore and confirm each selected feature is helpful for prediction, we conduct experiments with seven models, each of which uses a feature combination. The first model is constructed with the features of patient information, and we gradually add the features of blood test item as model features to construct the subsequent models. Table 1 shows the experimental results, indicating that the performances of the models increase as more features are used by the model. Moreover, once the model uses all the eight features, the AUC could be 0.799 when using the data 10 days before the surgery as the data source, meaning that the selected features are important for the prediction of survival after liver transplantation.

**Table 1:** Model performances with different feature combinations. We use RF to identify the top eight features, and conduct experiments to evaluate model performances with different feature combinations.

Model	Feature	AUC
1	BMI, age	$0.550\pm 0.079$
2	BMI, age, Na	$0.658\pm 0.176$
3	BMI, age, Na, Lymphocyte	$0.670\pm 0.157$
4	BMI, age, Na, Lymphocyte, INR	$0.742\pm 0.137$
5	BMI, age, Na, Lymphocyte, INR, WBC	$0.738\pm 0.157$
6	BMI, age, Na, Lymphocyte, INR, WBC, Platelets	$0.783\pm 0.157$
7	BMI, age, Na, Lymphocyte, INR, WBC, Platelets, Mg	$0.799\pm 0.138$

Once the feature selection process and pre-processing are completed, we use the selected features to learn a predictive model. This work proposes to use RF to construct the predictive model, and we compare RF with other alternatives by using patient basic information and blood test data from day 1 to day 9 before surgery as our final data source. Note that this work proposes to use RF for two tasks, feature selection and the predictive model. We use the derivation set with 10-fold cross-validation to evaluate model performance and confirmed the generalization ability of the proposal model with temporal validation set. Besides, we compare the proposed method with eXtreme Gradient Boosting (XGBoost)^31^, logistic regression, and decision tree^32^. The results are presented in Table 2, which shows that RF yields the best AUCs on derivation and temporal validation sets. The AUC of 0.771 on the temporal validation set indicates that the proposed model achieves superior discrimination power than other alternatives in predicting postoperative survival. We conclude that the RF uses bagging approach to combine various decision trees, giving a base to perform well on imbalanced data set. XGBoost uses another ensemble learning approach, boosting, and it also works well in the experiment.

**Table 2:** Performance comparisons for different learning algorithms on derivation and temporal sets. The values in the cell of the top table are the mean and 1.96 standard deviations of 10-fold cross-validation. Random forest and XGBoost both work well, and RF outperforms XGBoost in AUC.

Performance on Derivation Set			
Method	AUC	Specificity	Sensitivity
RF	0.787 ± 0.185	0.955 ± 0.187	0.653 ± 0.334
XGBoost	0.782 ± 0.268	0.905 ± 0.335	0.729 ± 0.329
Decision Tree	0.576 ± 0.229	0.698 ± 0.349	0.517 ± 0.335
Logistic Regression	0.538 ± 0.273	0.717 ± 0.230	0.695 ± 0.250
**Performance on Temporal Set**			
**Method**	**AUC**	**Specificity**	**Sensitivity**
RF	0.771	0.815	0.5
XGBoost	0.759	0.796	0.5
Decision Tree	0.632	0.888	0.25
Logistic Regression	0.671	0.870	0.5

## Discussion

**Figure 1:**
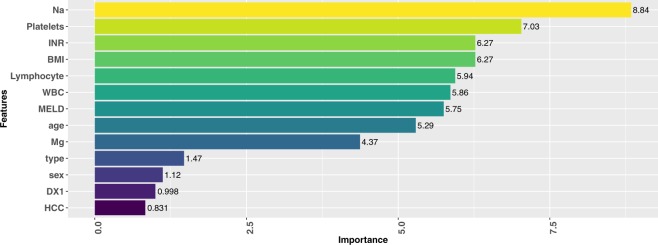
The top important features selected from RF with step-wise selection. These variables are verified by the physician. (DX1: reason for liver transplantation, type: type of hepatitis, HCC: Hepatocellular carcinoma).

In this study, we apply feature selection technique to select important features from the physiological measurement items. The top features presented in Fig. 1 are obtained from RF as well as step-wise selection, and the selected features include INR, Lymphocyte, Platelets, WBC, Mg, Na, age and BMI. Moreover, missing data in medical research is a common problem, and we propose an imputation method based on the characteristics of the features to deal with this problem. Next, detailed discussion regarding the findings is presented below.

Missing data in medical research is a common problem, and we propose an imputation method based on the characteristics of the features to deal with this problem, in which we use a conservative strategy to replace the missing values. We compare our proposed imputation method with the other alternatives, including imputation with mean, maximum, median and minimum, respectively. The results are presented in Table 3, indicating that the proposed method achieves the best predictive performance. The proposed method considers the characteristics of features, and we believe that is the main reason why the proposed method outperforms the other alternatives.

**Table 3:** Performance comparison of different imputation methods. The values in the cell are the mean and 1.96 standard deviations of 10-fold cross-validation on derivation set. The proposed method considers the characteristics of features, giving a base to outperform other alternatives.

Imputation Methods	AUC	Specificity	Sensitivity
Proposed Method	$0.787\pm 0.199$	$0.955\pm 0.187$	$0.632\pm 0.271$
Minimum	$0.785\pm 0.230$	$0.830\pm 0.327$	$0.749\pm 0.277$
Maximum	$0.779\pm 0.245$	$0.955\pm 0.187$	$0.667\pm 0.381$
Median	$0.756\pm 0.319$	$0.875\pm 0.347$	$0.730\pm 0.270$
Average	$0.753\pm 0.325$	$0.865\pm 0.296$	$0.754\pm 0.354$
Classification and Regression Tree	$0.736\pm 0.345$	$0.825\pm 0.321$	$0.750\pm 0.255$

**Figure 2:**
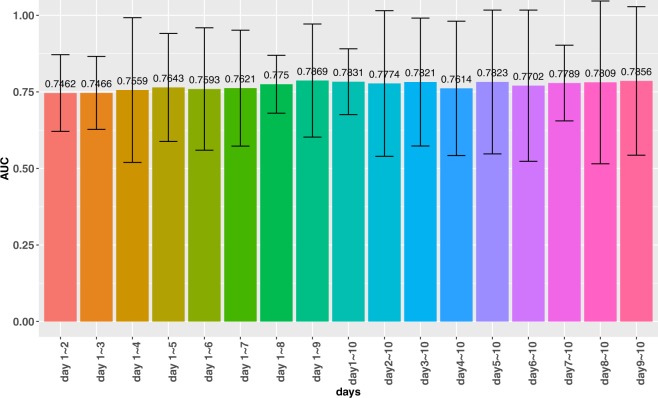
Experimental results with different range of days as the data source. The AUC increases as more training data are used in the model, and the data of day 1 to day 9 is the most important one.

This work uses the data 10 days before the surgery as the data source to construct the first model. Subsequently, we conduct experiments to find out which period of data before surgery has the most impact on the prediction results. The experiments use RF as the machine learning algorithm, and different ranges of data as the data sources to train different predictive models. The experimental results are presented in Fig. 2. The results show that the data from day 1 to day 9 before surgery is more important than the other ranges. This result conforms to the intuition as day 10 is the day farthest from the surgery in the range.

**Figure 3:**
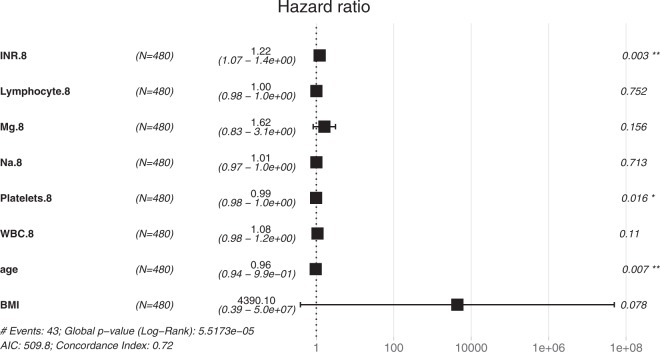
Hazard ratios (HR) from Cox proportional hazards model with the data of day 9. In the results, HR >1indicates an increased risk of death, and HR < 1 represents a decreased risk. The p-values of the variables show that INR, Platelets and age are significant features.

MELD score is a formula involving bilirubin, INR and creatinine. Thus, MELD score could be considered as a combination of the three features. To compare the performance impacts brought by the features, we use RF with MELD score and several features to learn a predictive model. The features used by the comparison model includes MELD score, hepatitis, HCC, DX1, age, gender, and BMI. The results are presented in the top of Table 4, indicating that our proposed model outperforms the alternative model for predicting postoperative survival. Besides, we apply cox proportional hazards model with the two combinations of features to perform survival analysis within 30 days and the results are listed in the bottom of Table 4. Both of them are statistically significant, but the features selected by RF achieves higher Concordance Index (0.85) than those used by MELD score. Moreover, the same experiments are applied to temporal validation set to investigate the generalization capability of our proposed model, and the results are presented in Table 5. The experimental results indicate that the features selected by RF could provide more discriminative capability than the features used by MELD score in predicting survival outcome after liver transplantation. Besides the above analysis, the hazard ratios (HR) from cox proportional hazards model are presented in Fig. 3, which only shows the basic features and the blood test data of day 9 owing to the limit of paper length. Significant features comprise INR, Platelets and age, which conform to the bedside experience of the domain expert.

**Table 4:** Performance comparison of RF and survival analysis with different combinations of features. The experiments were conducted with 10-fold cross-validation. The features used in MELD model comprise MELD score, hepatitis, HCC, DX1, age, gender, and BMI, whereas the features selected by RF are the top eight features identified by RF.

RF Model on Derivation Set			
Features	AUC	Specificity	Sensitivity
Features selected by RF	0.787 ± 0.185	0.955 ± 0.187	0.653 ± 0.334
Features used by MELD	0.596 ± 0.315	0.707 ± 0.302	0.720 ± 0.395
**Survival Analysis on Derivation Set**			
**Features**	**Concordance Index**	**Likelihood ratio test**	**Wald test**
Features selected by RF	0.85	p-value = 6e-08	p-value = 2e-05
Features used by MELD	0.695	p-value = 4e-04	p-value = 8e-05

**Table 5:** The effect of features on model performance in temporal validation data set. We applied RF with two combinations of features to the temporal validation set, and the experimental results point out that the model could benefit from the features selected by RF.

RF Model on Derivation Set			
Features	AUC	Specificity	Sensitivity
Features selected by RF	0.771	0.815	0.5
Features selected by MELD	0.681	0.703	0.5

The medical data used in this study is imbalanced as presented in most medical studies. We propose to use RF^29^ to construct the predictive model, which provides not only accurate performance, but also the capability of dealing with imbalanced data. This is because RF uses a technology called bagging that can reduce or mitigate bias for imbalance data^33–35^. Bagging approach uses bootstrap sampling technique to sample enormous sub-samples with replacement from the initial data set, each of the sub-samples is used to train a predictive model. In RF, each model is a classification and regression tree (CART)^32^, which is a decision tree algorithm. The final model is obtained by averaging all these models, and majority vote rule is a typical approach in determining the final results. The bagging approach provides a way to eliminate bias caused by unstable models. The experimental results indicate that RF works well on imbalanced data used in this study.

In conclusion, the analysis of experimental results presents two findings. First, among these important features, most of the features are blood test items and have been clinically proven that those features have a certain impact on survival outcome after liver transplantation except Mg. Our experimental results show that Mg is also an important feature which has impact on survival outcome after liver transplantation. Second, the experimental results show that RF is robust on imbalanced data. Most medical data sets are characterized by imbalanced property, and many medical applications are interested in the risk factors that lead to the results. Thus, RF is a very good machine learning model in medical domain.

Although previous research has indicated that patients who have undergone orthotopic liver transplantation may be a group especially predisposed to hypomagnesaemia^36^, the domain experts pointed out that Mg has not been used clinically to predict survival outcome after liver transplantation. However, blood magnesium ion concentration indeed is a very important electrolyte. If the patient has malabsorption or used diuretics, he/she would be considered as being in a high-risk group for hypomagnesemia. When the blood magnesium ion concentration is too low, it would directly affect the recovery of many other electrolytes. Moreover, the previous research has indicated that Mg has a direct relationship with heart function^37^, which associates Mg with mortality^38,39^, such as the association between hypomagnesemia and fatal cardiac arrhythmia^40^. Thus, another benefit of using data-driven approach to devise a predictive model is that one may discover the factors that are not directly related to the organs we are focusing on.

In summary, this work considers the characteristics of features to propose an imputation method to deal with missing values, and the results point out that the proposed method works well. Central to this study is using machine learning to predict short-term survival which can detect the high risk patients in early phase after liver transplantation, and discover important factors that are essential in liver transplantation, in which we argue machine learning could help the physicians make decisions. Once the higher risk patients are identified by the model, several treatment options could be given to these patients. For example, the immunosuprpesion drug should be admitted earlier or in relative high concentration, to avoiding the trigger of acute rejection and causing the vulnerable complications, such as acute kidney injection, and secondary bacterial infection.

## Methods

**Figure 4:**
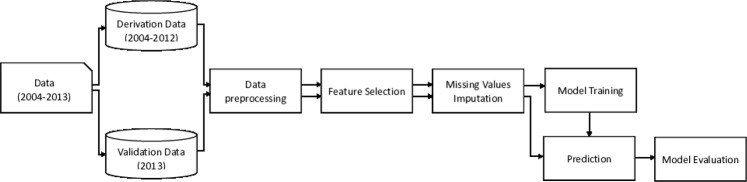
Experimental flow. The experimental flow comprises data pre-processing, feature selection, imputation of missing values, model training and evaluation. The purpose of training data is for model training, whereas testing data is used for model evaluation.

In this study, the data in the experiments was collected by liver transplantation ICU of Chang Gung Memorial Hospital, Linkou and has been approved by institutional review board (IRB) of Chang Gung Memorial Hospital with case number 103-6018B. All the data and methods were performed in accordance with the relevant guidelines and regulations by IRB of Chang Gung Memorial Hospital. Additionally, this work is a retrospective study, and the IRB waived the need for informed consent. The patient data ranges from January, 2004 to December, 2013, and the number of data records is approximately two million. We divide the whole research process into several stages as shown in Fig. 4.

The first stage is data pre-processing, including two steps: (1) Data cleaning: we follow the suggestion of domain experts to clean the data, including unifying the name of test items, processing the extreme values, removing the duplicated data, and so on. (2) Defining survival time: because our objective is to predict postoperative survival, we use the "Postoperative survival days” in the patient’s personal information as an indicator to define survival time.

The second stage is feature selection. In this study, we purpose to use RF to select features, and the goal is to select important features from the whole blood tests. A model with enormous features may suffer from over-fitting problem. Thus, it is expected that the final model could benefit from feature selection. The third stage is to perform imputation of missing values to replace missing values with meaningful values. In this work, we separate the data into derivation set and temporal validation set based on time information. Table 6 shows patient characteristics in derivation and temporal validation sets. The purpose of derivation set is to train a predictive model, while temporal validation set is used for model evaluation. Therefore, in the fourth stage, we use derivation set to train the RF model. Finally, we use temporal validation set to evaluate the model.

**Table 6:** Patients’ characteristics at 9 days before the surgery in derivation and temporal validation sets. The characteristics comprise basic information, blood test, preoperative status and other details. Moreover, statistical tests are applied to the data. *Mann-Whitney U Test were performed for continuous data, and Pearson Chi-Squared Tests for categorical data between groups comparison.

Predictor candidates		Derivation set (year = 2004–2012) (${\boldsymbol{n}}{\boldsymbol{=}}{\bf{480}}$)				Temporal validation set (year = 2013) (${\boldsymbol{n}}{\boldsymbol{=}}{\bf{58}}$)			
		${\boldsymbol{n}}$	Survival ${\boldsymbol{(}}{\boldsymbol{n}}{\boldsymbol{=}}{\bf{437}}{\boldsymbol{)}}$	Death ${\boldsymbol{(}}{\boldsymbol{n}}{\boldsymbol{=}}{\bf{43}}{\boldsymbol{)}}$	p-value*	${\boldsymbol{n}}$	Survival ${\boldsymbol{(}}{\boldsymbol{n}}{\boldsymbol{=}}{\bf{54}}{\boldsymbol{)}}$	Death ${\boldsymbol{(}}{\boldsymbol{n}}{\boldsymbol{=}}{\bf{4}}{\boldsymbol{)}}$	p-value*
**Demographic data, mean(sd)**									
1	Gender, n(%)				1.0				1.0
	Male	358	326 (74.60)	32 (74.42)		43	40 (74.07)	3 (0.75)	
	Female	122	111 (25.40)	11 (25.58)		15	14 (25.93)	1 (0.25)	
2	Age	480	52.69 (9.18)	47.93 (15.29)	0.0982	58	52.63 (10.48)	55.25 (7.72)	0.6896
3	BMI	470	24.52 (3.90)	87.00 (39.65)	0.3626	57	26.25 (4.26)	24.05 (2.30)	0.2918
4	Survival days	480	1411.25 (1014.81)	12.14 (7.04)	<2.2E-16	58	172.70 (76.51)	11.75 (11.95)	0.00097
**Blood test of day 9, mean (sd)**									
5	INR	470	1.73 (0.85)	2.51 (2.74)	4.724E-06	56	1.58 (2.04)	0.56 (0.56)	0.07164
6	Lymphocyte	467	22.33 (12.31)	18.55 (13.33)	0.02734	57	22.14 (11.40)	18.23 (8.87)	0.5219
7	Mg	415	1.68 (0.24)	1.65 (0.33)	0.1257	50	1.69 (0.26)	1.55 (0.23)	0.3133
8	Na	268	137.06 (5.82)	138.17 (10.12)	0.9906	8	123.28 (5.91)	122.14 (2.32)	0.9908
9	Platelets	476	79.21 (49.16)	63.39 (35.46)	0.0814	57	81.64 (68.08)	85.08 (18.66)	0.2811
10	WBC	476	4.86 (3.26)	5.99 (3.79)	0.03277	57	4.83 (5.16)	6.67 (3.56)	0.1378
**Preoperative status, mean(sd)**									
11	MELD score	480	17.84 (8.90)	24.21 (9.36)	1.617E-05	58	16.69 (10.04)	29.75 (11.90)	0.021
12	Hepatitis, n(%)				0.1996				0.2852
	Nil	83	71 (16.25)	12 (27.91)		19	16 (29.63)	3 (0.75)	
	Hepatitis B Virus (HBV)	277	253 (57.89)	24 (55.81)		22	21 (38.89)	1 (0.25)	
	Hepatitis C Virus (HCV)	96	90 (20.59)	6 (13.95)		16	16 (29.63)	0 (0)	
	Dual	24	23 (5.27)	1 (2.33)		11	1 (1.85)	0 (0)	
13	HCC, n(%)				0.004467				0.2002
	No	264	231 (52.86)	33 (76.74)		33	29 (53.70)	4 (1.0)	
	Yes	216	206 (47.14)	10 (23.26)		25	25 (46.30)	0 (0)	
14	DX1, n(%)				0.1942				—
	Virtual hepatitis	396	363 (83.26)	33 (76.74)			43 (79.63)	2 (0.50)	
	Alcoholic cirrhosis	35	32 (7.34)	3 (6.98)			6 (11.11)	1 (0.25)	
	Wilson’s disease	6	4 (0.92)	2 (4.65)			1 (1.85)	0 (0)	
	Primary Biliary cirrhosis	7	7 (1.61)	0 (0)			0 (0)	0 (0)	
	Biliary atesia	2	1 (0.23)	1 (2.33)			0 (0)	0 (0)	
	Fulminiant hepatitis	4	3 (0.69)	1 (2.33)			0 (0)	0 (0)	
	Secondary Biliary cirrhosis	1	1 (0.23)	0 (0)			0 (0)	0 (0)	
	Other malignancy	2	2 (0.46)	0 (0)			0 (0)	0 (0)	
	Others	26	23 (5.26)	3 (6.98)			4 (7.41)	1 (0.25)	
**Other details, mean(sd)**									
15	Graft-recipient weight ratio (GRWR, %)	387	1.01 (0.24)	1.03 (0.48)	0.4077	53	0.94 (0.2)	1.02 (0.23)	0.5004
16	Liver weight	413	633.15 (146.82)	520.61 (134.30)	$4.163E-05$	50	644.68 (129.73)	620 (256.60)	0.8381
17	Types of liver transplant, n(%)				0.03325				0.7745
	Living donor	414	382 (87.41)	32 (74.42)		54	51 (94.44)	3 (0.75)	
	Deceased donor	66	55 (12.59)	11 (25.58)		4	3 (5.56)	1 (0.25)	

### Data pre-processing

The exported data was presented in comma separated values (CSV) format, and we used R (version 3.6.1) to process the data and build the predictive model. The data pre-processing involves two tasks in this study, and they are described in the following sections.

#### Data cleaning

This work focuses on the prediction of postoperative survival, so it is natural to use the data records before the surgery to construct a predictive model. We retain the data records 10 days before the surgery for analysis. Several data cleaning steps are used to make the data suitable for the subsequent analysis, and these steps are listed below. The final items used in the experiments are listed in Table A.1 of our previous work regarding the prediction of acute allograft rejection after liver transplantation^41^.

- Retaining the data within 10 days: The original data comprises the patient’s postoperative records and the records from long-term follow-up study. The goal is to predict postoperative survival, so we only kept the data of 10 days before the surgery.
- Removing the items of urine test: Urinalysis results are less accurate than blood tests, so we used blood test data in the model without using urine test data in this work.
- Removing duplicated measurement: When multiple measurement records were present in the examination results, we used the average value to represent the measurement value.
- Calculating BMI: We used Eq. (1) to calculate BMI based on the patient’s height and weight.

1 $$\,{\rm{BMI}}=\frac{{\rm{Weight}}}{{{\rm{Height}}}^{2}}$$

#### Data labeling

In this study, we retain those data records 10 days before the surgery for analysis and used the “Postoperative survival days” in the patient’s personal information as an indicator to define survival. To exclude factors that affect modeling such as quality of life, diet and others, we focus on short-term survival prediction. In addition, liver function would not recover until 30 days after liver transplantation, so we define the survival time of more than 30 days as “Survival” and others as “Non-survival”. Once the data labeling is completed, the data set comprises 538 patients, including 491 survival patients and 47 non-survival patients, respectively. The causes of death for non-survival patients are listed in Table 7.

**Table 7:** The causes of death for the short-term survival patients in derivation and temporal validation sets.

Cause of death	Derivation set (n = 43)	Temporal validation set (n = 4)
Acute Cellular Rejection	9	—
Acute Humeral Rejection	5	1
Primary non function	3	—
Sepsis	13	2
Cardiopulmonary	7	1
Complication	—	—
Small for size graft	3	—
Others	3	—

### Feature selection

RF combines "bagging” technique as well as random subspace method^42^ to construct enormous decision trees. It is important to construct uncorrelated decision trees during the learning process, and random subspace method is an ensemble learning method that applies to features to reduce the correlation between the trees by using a random sample of features to construct each decision tree. As RF relies on a collection of decision trees to make the prediction, it provides a way to estimate feature performance from all the decision trees by measuring the impact of each feature on accuracy of the model. The idea is to permute the values of a feature $i$, and test its importance by measuring how much the permutation decreases the accuracy of the model. For important features, the permutation would significantly decrease model performance. In contrast, permuting unimportant ones should have little impact on model performance. Once the importance scores are available, one could use these scores to rank the features.

### Imputation of missing values

Missing values are always present in the data records, which may come from human errors or the patient did not perform some tests. Ignoring these values may cause the model to be unstable. As a consequence, we propose a method to replaces missing values with reasonable values. The proposed approach is a conservative strategy, and the imputation is based on feature characteristics and domain knowledge. The steps are listed as follows:

- STEP 1 - Stratifying the data by MELD score: Based on the MELD score, we divided all data into several groups, including 1–9, 10–19, 20–29, 30–39, and 40 or more.
- STEP 2 - Dividing features into three categories: The three categories are listed below:
  - A features: The higher the value, the worse the prognosis. For example, age and INR.
  - B features: The lower the value, the worse the prognosis. For example, Na.
  - C features: The value is too low or too high, the worse the prognosis. For example, WBC.
- STEP 3 - Replacing the missing values: Different rules for replacing missing values are applied based on different groups of MELD scores and different categories of features.
  - If missing value belongs to A features, we will replace this value with the maximum for that group.
  - If missing value belongs to B features, we will replace this value with the minimum for that group.
  - If missing value belongs to C features, we will replace this value with the average for that group.

### Model construction

Since the scales of physiological measurement are quite different, we take the nature log of them first. In the previous steps, the important items obtained from feature selection are INR, Lymphocyte, PT, Platelets, WBC, Mg, Na, age and BMI. However, according to expert’s experience, INR and PT represent the same measurement. To prevent from bias, we only use INR to construct the model. Next, we separated the data into derivation set and temporal validation set. The models are trained by RF, XGBoost, Decision Tree and Logistic Regression algorithms, and each algorithm made ten different results. Notably, we use AUC as the performance metric because of the data imbalance.

One of the limitations for this work is that we do not apply external validation to our proposed model. This is our initial step that attempts to use machine learning to develop a predictive model for the prediction of predict short-term survival after liver transplantation. To objectively assess our proposed model, we use a systematic approach to develop the model, and separate the data into derivation set and temporal validation set based on time information. We believe that the findings in this work are useful for other researchers, and applying external validation to our proposed model is our future work.

## Data Availability

The data that support the findings of this study are available from Chang Gung Memorial Hospital, Linkou, Taiwan but restrictions apply to the availability of these data, which were used under license for the current study, and so are not publicly available. Data are however available from the authors upon reasonable request and with permission of Chang Gung Memorial Hospital, Linkou, Taiwan.
